# Effect of Laser Shock Peening on Fretting Fatigue Life of TC11 Titanium Alloy

**DOI:** 10.3390/ma13214711

**Published:** 2020-10-22

**Authors:** Xufeng Yang, Hongjian Zhang, Haitao Cui, Changlong Wen

**Affiliations:** College of Energy and Power Engineering, Nanjing University of Aeronautics and Astronautics, Nanjing 210016, China; yangxufeng@nuaa.edu.cn (X.Y.); cuiht@nuaa.edu.cn (H.C.); changlongwen91@163.com (C.W.)

**Keywords:** fretting fatigue, laser shock peening, laser power density, TC11 alloy

## Abstract

The purpose of this paper is to investigate the performance of laser shock peening (LSP) subjected to fretting fatigue with TC11 titanium alloy specimens and pads. Three laser power densities (3.2 GW/cm^2^, 4.8 GW/cm^2^ and 6.4 GW/cm^2^) of LSP were chosen and tested using manufactured fretting fatigue apparatus. The experimental results show that the LSP surface treatment significantly improves the fretting fatigue lives of the fretting specimens, and the fretting fatigue life increases most when the laser power density is 4.8 GW/cm^2^. It is also found that with the increase of the laser power density, the fatigue crack initiation location tends to move from the surface to the interior of the specimen.

## 1. Introduction

Fretting is usually recognized as a kind of near-surface damage arising from the relative slip between the two mating components that are clamped together with a normal force [1]. If the relative slip of contact surfaces is caused by the cyclical stress load of one of the contact components, then the crack may nucleate and continue to propagate to fatigue failure, which is called fretting fatigue. Fretting is a problem in many aerospace applications such as airframe lap joints and the dovetail attachment between the engine blade and disk [2]. In fact, fretting or fretting fatigue has been identified as one of the costliest sources of in-service damage in the US Air Force [3]. Considering that the original design configuration of many components is difficult to change, the research on surface protection techniques of fretting fatigue has been developed since the 1960s, and some techniques have been applied successfully [4]. The principle of most surface protection techniques, such as coating and shot peening, is to introduce residual compressive stress into the contact surface, reduce the friction coefficient of the contact surface, and improve the hardness and wear resistance of the contact surface [5,6,7,8,9,10]. As an advanced surface treatment, laser shock peening (LSP) which induces the deep compressive residual stresses beneath the contact surfaces has been proved to be significantly beneficial to improve the fretting fatigue lives [11].

**Figure 1:**
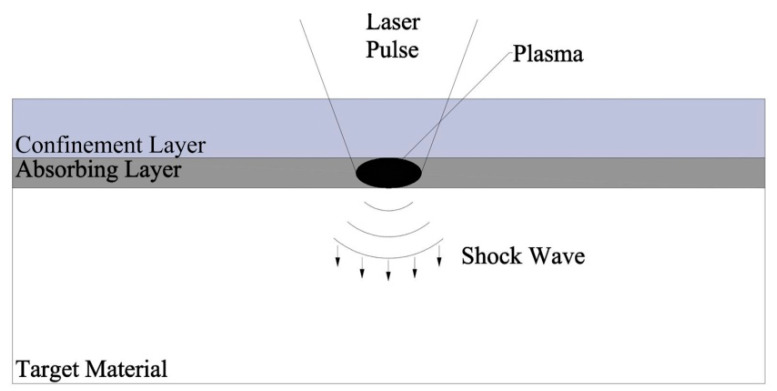
Schematic of the laser shock peening (LSP).

The simple process of LSP is shown schematically in Figure 1. The region to be treated is covered by an absorbing layer, over which is the confinement layer which is usually used as running water [12]. A high-energy laser pulse is directed on to the surface, which passes through the confinement layer and is absorbed by the absorbing layer which is usually used as a black coating, metal sacrifice layer or black tape [13,14,15]. Then, the plasma is formed over the absorbing surface. The expansion of the plasma is constrained by the running water, which drives a shockwave into the material, and this shockwave causes the plastic deformation and residual stresses near surface region. The relevant research results show that under certain conditions, there is a critical value for the thickness of the absorbing layer. When the thickness exceeds the critical value, the non-vaporized part will cause the loss of shock wave. When the thickness is lower than the critical value, the unabsorbed laser energy will vaporize the metal surface, thus forming rough impact pits and reducing the impact effect [16].

It is believed that compared with the conventional shot peening, LSP can produce compressive stresses to greater depths and has better resistance to fretting fatigue. In fact, the fretting fatigue process can also induce residual compressive stress in the surface layer of materials, and with the increase of the fatigue loading cycle, the residual stress levels presents a trend of first increase and then decrease [17]. However, it is found that fretting fatigue loading on the experimental samples treated with LSP can cause significant stress relaxation extending to a certain depth [18], and this interesting phenomenon may indicate the possibility that the fretting fatigue process may reduce the effect of the LSP. In addition, another interesting result has been found in work on the effect of laser peening and shot peening on fretting fatigue of TC4 titanium alloy [19]. In some fretting fatigue load conditions, the crack initiation points appear in the subsurface layer where the compensated tensile stress is max instead of the surface. It is believed that the reason for the fatigue failure of the specimens is the compensated tensile stress rather than the fretting effect. This result shows that LSP can significantly reduce the impact of fretting fatigue damage, but at the same time, the compensating tensile stress caused by the introduction of residual compressive stress layer should be considered. The superposition of compensating tensile stress and external load will increase the local stress level, which will lead to failure.

Although most of the relevant researches successfully attribute the improvement of fretting fatigue life to the introduction of residual compressive stress influencing layer, few researches care about the influence of the parameters of the laser shock itself. In fact, there are many parameters affecting the fretting fatigue resistance of LSP, including the overlap ratio of laser spots, laser energy, laser spot diameter, impact times, laser pulse duration and so on [20]. Thus, different combination of these parameters may cause different residual stress influencing layers and different effect on fretting fatigue resistance. 

Laser power density *I*_0_ can be described as Equation (1) [21], where *E* is the pulse energy, *R* means the radius of the laser spot and *τ* is the laser pulse duration. It is recognized that a better residual compressive stress distribution can be obtained by selecting the appropriate laser power density [21,22], and an excessive laser power density will lead to indentation on the surface of the material, and even ablation of the absorbing layer [23,24].

(1) $$I_{0}=\frac{E}{4\pi R^{2}\tau }$$

Laser pulse duration *τ* is used to measure the time distribution characteristics of pulse laser, which has an important influence on the depth of the residual stress influence layer [25]. It is found that with the increase of pulse duration, the duration of shock wave pressure increases, and the plastic deformation of the material intensifies, which is beneficial to improve the surface residual stress after impact strengthening [26,27,28,29]. However, when the pulse duration width exceeds a certain value (about 10 ns) [28], the improvement effect of residual stress field in depth direction is relatively stable, and when the pulse duration width continues to increase to 24 ns [27], the surface residual compressive stress decreases. In addition, the excessive pulse duration may lead to the excessive loss of absorbing protective layer and laser ablation of surface materials [27].

In this paper, it was desired to investigate the influence of the laser power density for the effect of laser shock peening on fretting fatigue of TC11 titanium alloy, and three laser power densities of LSP were chosen and tested using manufactured fretting fatigue apparatus.

## 2. Experiments 

### 2.1. Specimen Materials 

The material used here, both for specimens and fretting pads, was TC11 (Ti-6.5Al-1.5Zr-3.5Mo-0.3Si) titanium alloy. TC11 titanium alloy is widely used in the China aviation field, and the chemical composition of this alloy (in wt.%) is given in Table 1 [30]. TC11 titanium alloy is a kind of α + β type heat-resisting titanium alloy with an outstanding combination property. It is mainly used to manufacture compressor blades and disks in aero-engines. The heat treatment was double annealing, 950 °C/2 h air cooled and 530 °C/6 hair cooled. The material properties of this alloy at room temperature are listed in Table 2 [30].

### 2.2. Laser Shock Peening (LSP) Specimen and Procedure

**Figure 2:**
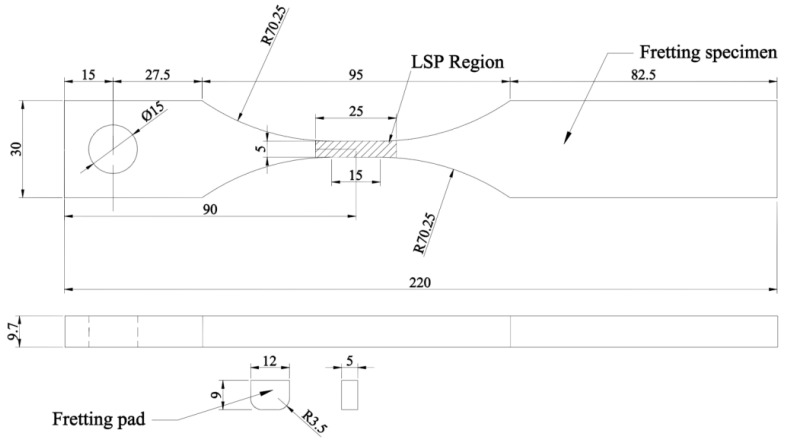
Main specimen and pad for fretting fatigue tests (mm).

All the main fretting specimens and the fretting pads were manufactured from the TC11 plate. The fretting pads were flat pads with rounded corners and loaded against the main “dog bone” specimen. The details of the main specimen and pad for fretting fatigue tests are shown in Figure 2. The LSP region, which means laser shock peening area, is a rectangular region of a size of 25 mm × 25 mm, and both two sides of the specimen are under LSP.

**Figure 3:**
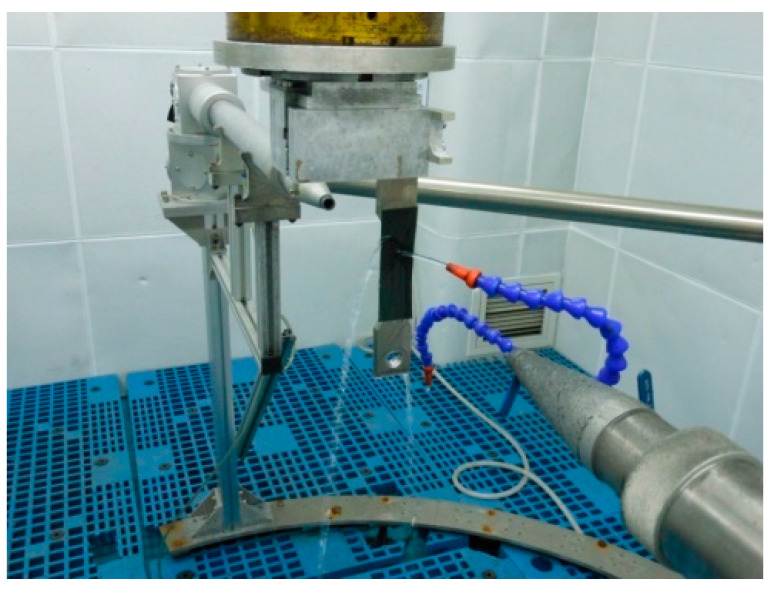
Picture of YD60-M165 laser system.

The LSP experiment was carried out by the YD60-M165 laser system with a wavelength of 1064 nm and laser pulse width of 20 ns (Figure 3). The surfaces of the test samples were covered with the black tape (0.1 mm thick), which was used as the absorbing layer for plasma initiation to protect the test surface from the thermal damage of high-temperature plasma. During the LSP, the test specimens were under a water bath, and the water layer with a thickness of about 1 mm was used as the confinement layer. During the experiment, the test piece moved in a presupposition route by the manipulator, and the laser beam was fixed and perpendicular to the sample surface all the time. 

**Figure 4:**
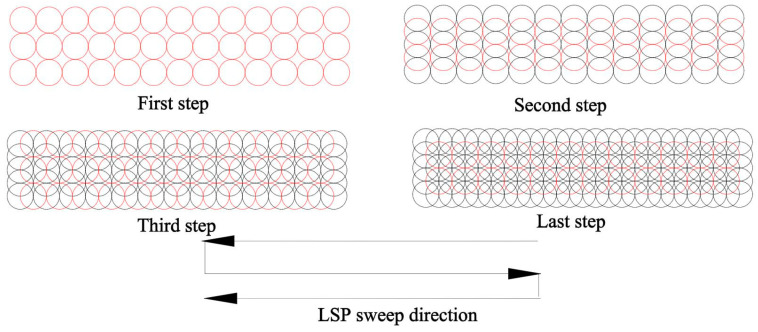
Diagram of the laser sweep steps and direction.

**Figure 5:**
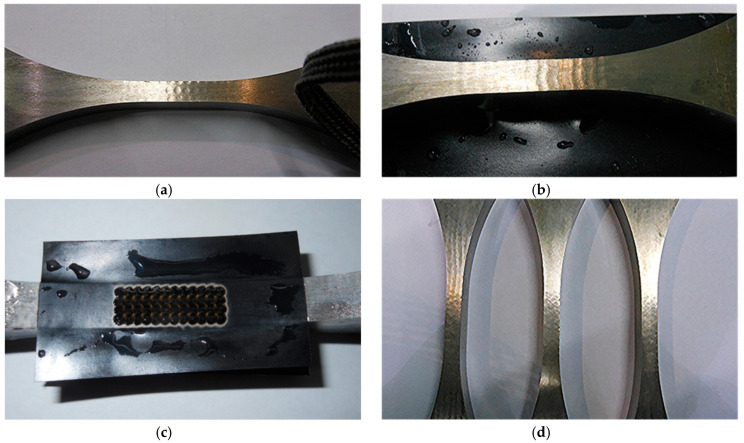
Surface condition after the LSP impacts. (**a**) First step. (**b**) Second step. (**c**) Third step. (**d**) Last step.

To guarantee the quality of LSP, multi LSP impacts were used, and the black tape was replaced after each impact to prevent the thermal damage of the sample surface because of the erosion of the black tape. The diameter of the laser spot was 2 mm and the detailed laser sweep steps and direction are shown in Figure 4, and the detailed surface condition after the LSP impacts is shown in Figure 5. The overlapping rate was 50% between two adjacent spots to ensure a good surface condition. 

The laser power densities chosen here were: 3.2 GW/cm^2^, 4.8 GW/cm^2^ and 6.4 GW/cm^2^. The details of the parameters of laser shock peening are listed in Table 3.

### 2.3. Surface Topography and Residual Stress

The surface topography of the untreated and LSP samples were carried out by using a non-contact 3D optical profilometer. The Z-direction scanning range of the profilometer is 0.1 nm to 10 mm. The measuring area of the surface topography was 4.5 mm × 3.4 mm, which was larger than the laser spot diameter used in this experiment. This measuring area could contain the typical characteristics of impact surface, and meet the requirements of surface topography and roughness measurement in this paper.

**Figure 6:**
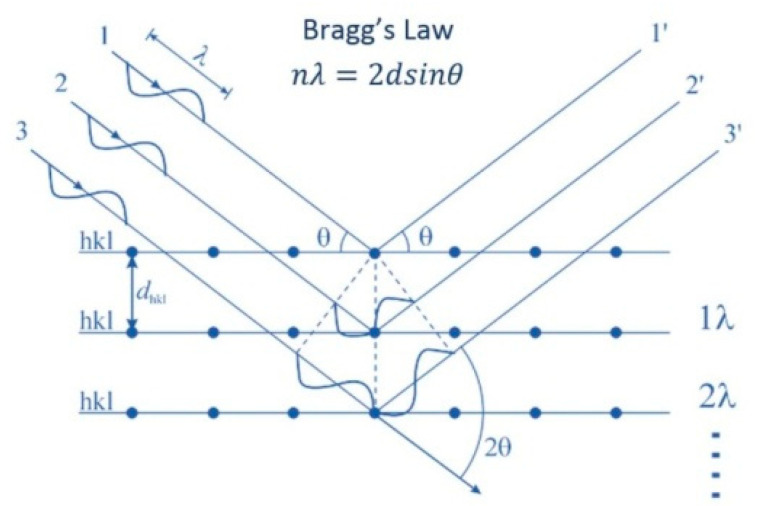
Principle of X-ray residual stress measurement.

In this paper, the X-ray diffraction (XRD) method is used to test the residual stress, and the test equipment used here is a proto LXRD X-ray diffractometer. The basic principle of X-ray residual stress measurement is shown in Figure 6. 

When the surface of the metal, which has residual stress, is measured, the crystal lattice spacing *d* changes. When the X-ray with wavelength *λ* is incident at angle *θ*, the corresponding diffraction angle 2*θ* is measured. The relationship between the optical path difference of X-ray and wavelength *λ* is as follows:

(2) 2dsinθ=nλ

**Figure 7:**
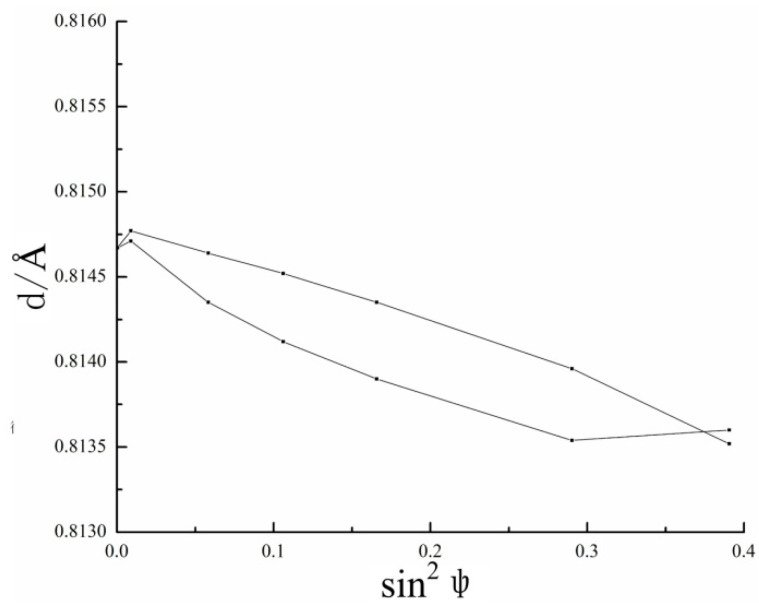
Diagram of the d–sin^2^ψ.

The radiation length used in this paper was 1.5418 Å, the diffraction type was Cu-K-Alpha, the diffraction plane (h, k, l) was (2, 1, 3), the crystal structure was HCP and the diffraction angle were 142 degrees. The principle of residual stress measurement used here was the sin^2^ψ method, and the range of the ψ were −39 to +39 degrees. The spring constants used here for the stress calculations were: S_1_ = −2.97 × 10^−6^ MPa^−1^, S_2_ = 23.78 × 10^−6^ MPa^−1^. The typical example of the *d* (interplanar spacing) − sin^2^ψ diagram was shown in Figure 7.

**Figure 8:**
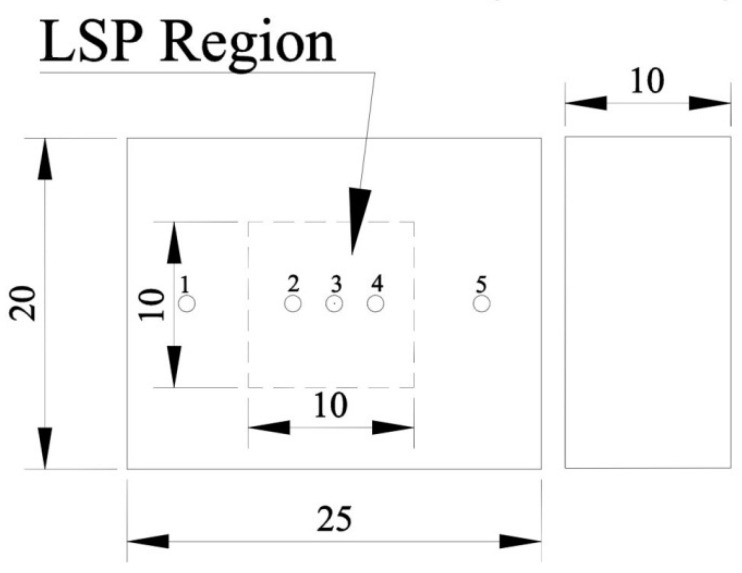
Test sample for surface residual stress (mm).

For the convenience of the measurement, the test sample used here is shown in Figure 8. All the five test points are on the midline of the test sample, and the test point 3 is at the center of the LSP region. The LSP region, which contains test points 2, 3, and 4, is in the center area of the test sample. Test point 1 and 5 are out of the LSP region and reflect the residual stress of the untreated area. 

Due to the limited penetration of X-ray, the residual stress on the surface of the material could only be measured. In view of the residual stress inside the material, the electrolytic polishing method was used to peel off the surface material layer by layer, and the polishing fluid was 24% HNO_3_, 14% HF and 62% H_2_O (volume ratio). The diameter of the etched area was 10 mm, and each period of electro polishing was 15 s, and the total polishing measurement was taken five times. The thickness of the material layer removed by etching was measured after each time of electro polishing. Then, the residual stress inside of the material could be measured by proto LXRD X-ray diffractometer.

### 2.4. Fretting Fatigue Test

**Figure 9:**
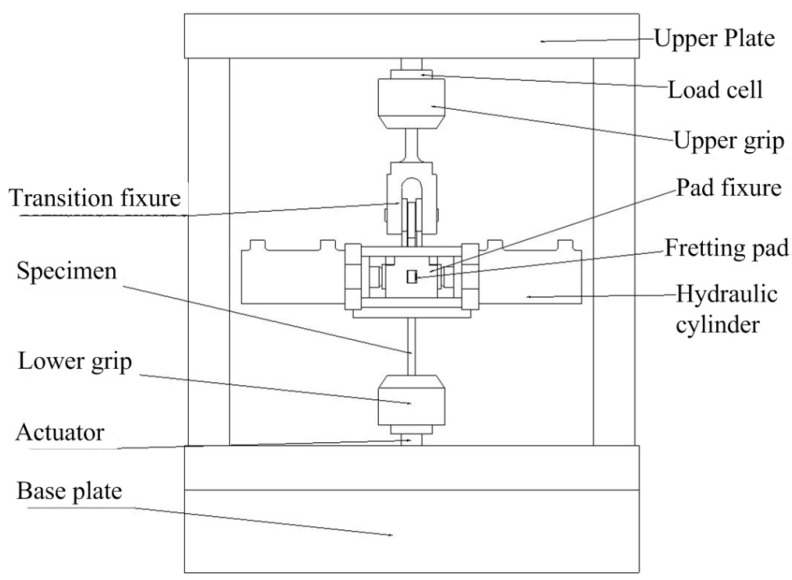
Diagram of the fretting fatigue test rig.

To conduct the fretting tests, the fretting fatigue test rig, which is constructed by an ordinary load frame with an additional pad fixture and a hydraulic cylinder, was used. A schematic diagram of the fretting fatigue rig is shown in Figure 9.

**Figure 10:**
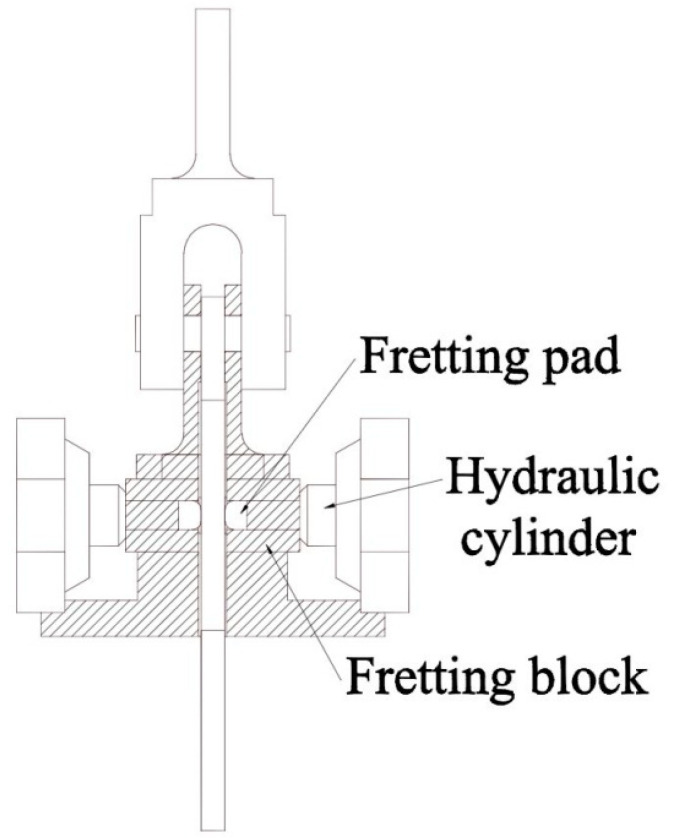
Diagram of the pad fixture.

The bottom of the main specimen is held by the moveable lower grip, while the top of the specimen is connected with the pad fixture and the transition fixture, which is held by the fixed upper grip, through a bolted connection. The moveable lower grip is mounted to the load cell and to a hydraulic actuator capable of applying fatigue loads to the specimen up to 50 KN. The two fretting pads are then held by the pad fixture, which is shown in Figure 10, so that the pads can be clamped by the hydraulic cylinder and contacted with the specimen. The hydraulic cylinder, which produces the normal load to the specimen, is fixed on the pad fixture with four long bolts, and to keep this normal load constant through the whole test, an accumulator is connected to the hydraulic line. The normal load is then calculated from the hydraulic pressure.

**Figure 11:**
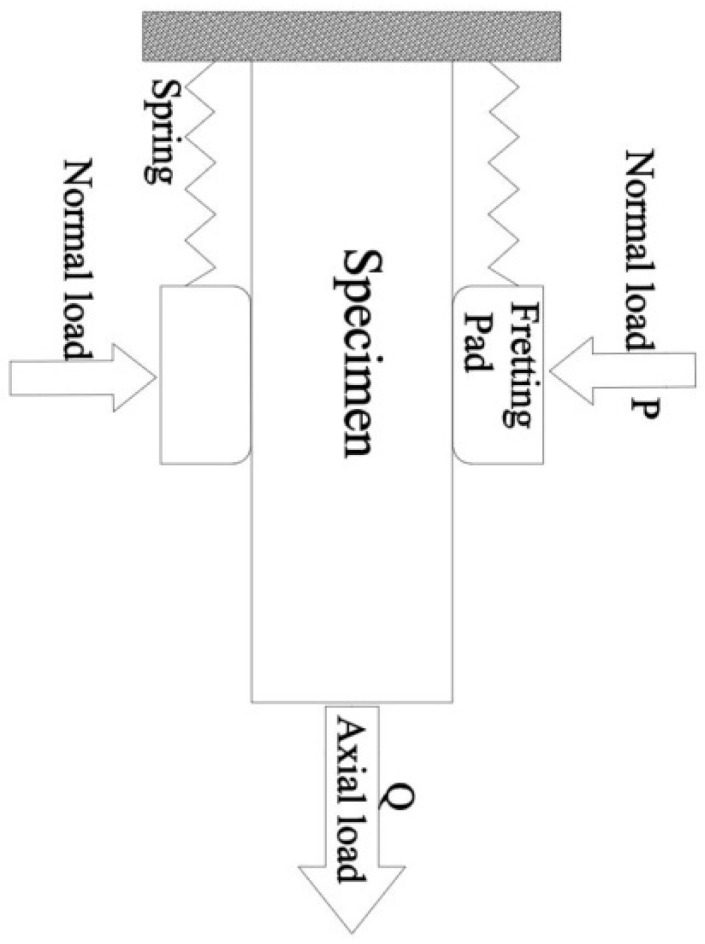
Simple load configuration of the test.

**Figure 12:**
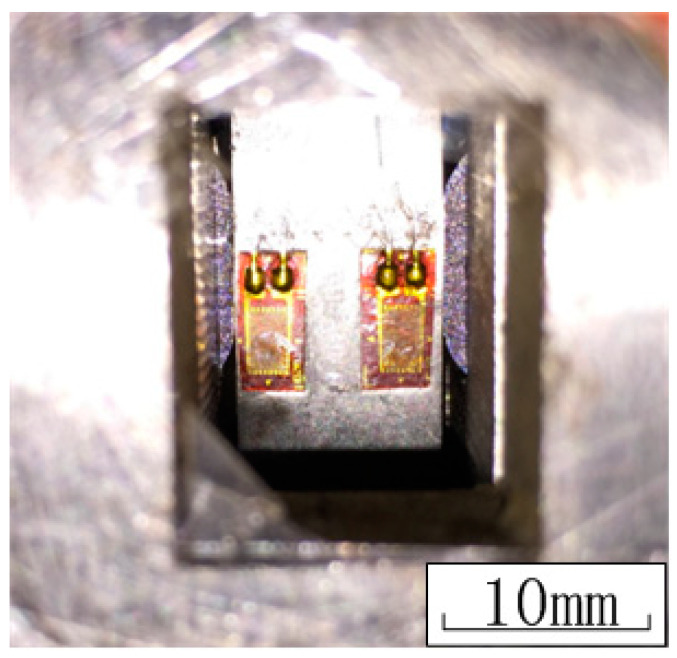
Picture of the strain gauges.

The static normal force, *P*, was first applied to the fretting pads. Then the cycle tensile load varying between *Q_min_* and *Q_max_* was applied to the specimen. Figure 11 shows a simple load configuration of the test. Table 4 reports the experimental parameters of the tests. Eight fretting fatigue tests were carried out and the lives of the crack initiation were recorded. Strain gauges bonded on both sides of the main specimen (Figure 12) were used to monitor the initiation of the crack. Once the crack occurred, the strain curves would rise immediately until the strain gauges were broken. At the same time, the hole in the middle of the pad fixture could help us to observe the crack initiation.

## 3. Results and Discussion

**Figure 13:**
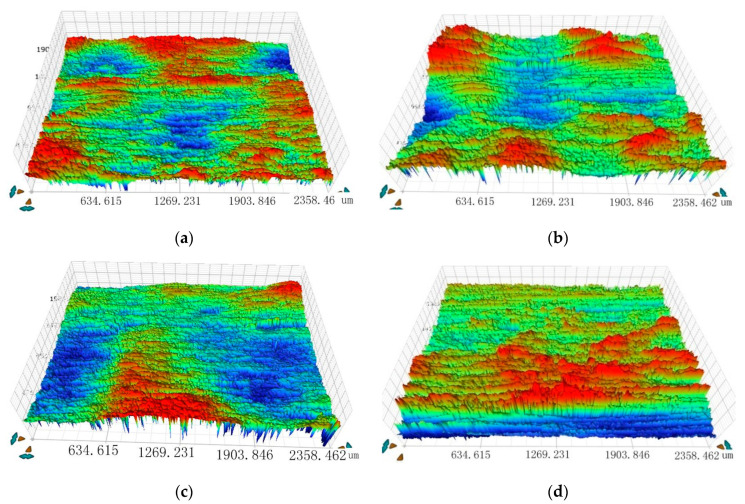
Three-dimensional surface morphologies of the main specimens. (**a**) 3.2 GW/cm^2^ (**b**) 4.8 GW/cm^2^ (**c**) 6.4 GW/cm^2^ (**d**) untreated.

Three-dimensional surface morphologies and surface roughness of the main specimens with different laser power densities are shown in Figure 13 and Table 5 respectively. There are obvious pits on the surface of the LSP area, and there are convex parts in the adjacent areas of the two pits, and the surface of the specimen is slightly uneven. In this experiment, the overlap rate of adjacent spots was 50% so that all the surfaces of the test area were affected by LSP, and some of them were overlapped areas, where plastic deformation was relatively large. Compared with the untreated samples, the surfaces of the LSP samples have obvious pits and lager surface roughness. The surface roughness increases with the increase of laser power density, which means a larger laser power density may cause a worse surface condition.

Table 6 shows the results of the surface compressive residual stresses of the test samples in the laser power densities of 3.2 GW/cm^2^, 4.8 GW/cm^2^ and 6.4 GW/cm^2^, and the average surface compressive residual stresses of the treated part are −554 MPa, −650 MPa and −645 MPa respectively. It seems that with the increase of the laser power densities, the average surface compressive residual stresses will reach a peak value and change a little. Continuing to increase the laser power density will cause the peak pressure of shock wave to be too high, which will affect the surface quality of the material. In addition, the residual compressive stress on the surface of the material will be reduced to a certain extent due to the generation of a surface unloading wave.

**Figure 14:**
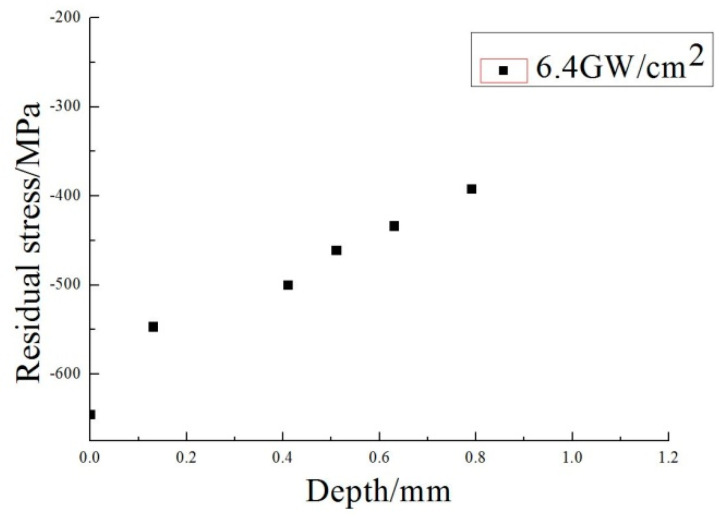
Distribution of the compressive residual stresses inside of the samples (6.4 GW/cm^2^).

Figure 14 shows the distribution of the compressive residual stresses inside of the samples in the laser power densities of 6.4 GW/cm^2^, and the residual compressive stress affects the depth of the layer by at least 0.8 mm.

**Figure 15:**
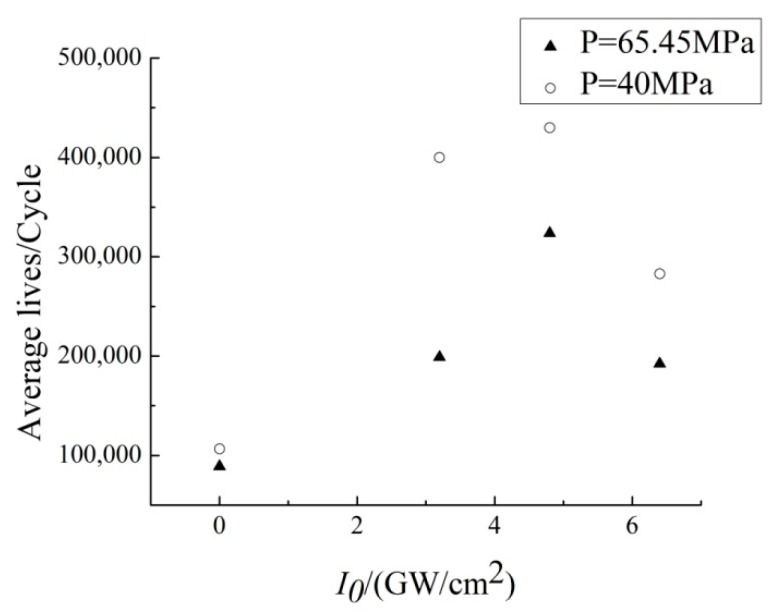
Average fretting fatigue life of TC11 with different laser power densities.

Table 7 and Figure 15 shows the fretting fatigue life and average fretting fatigue life of all the specimens respectively.

**Figure 16:**
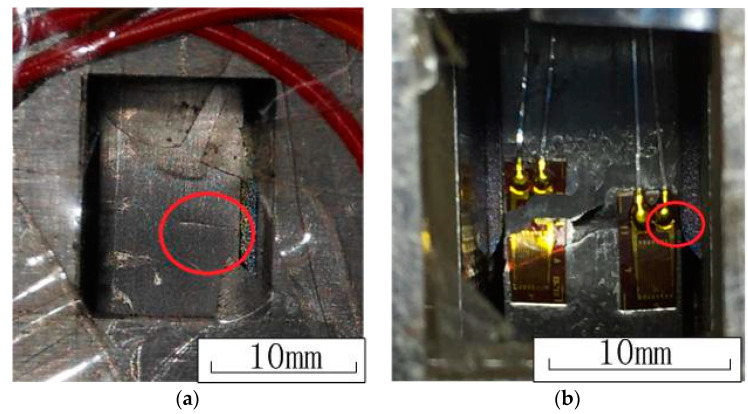
Pictures of the crack initiation and Specimen fracture. (**a**) Crack initiation. (**b**) Specimen fracture.

Compared with the untreated specimens, the fretting fatigue lives of LSP specimens have been significant increased, and the laser power density of 4.8 GW/cm^2^ shows the best effect to prolong the fretting fatigue life. As mentioned before, the compressive residual stress of the laser power density of 4.8 GW/cm^2^ and 6.4 GW/cm^2^ is similar, but the surface condition of the laser power density of 6.4 GW/cm^2^ is worse, which causes a shorter fatigue life. All the fatigue crack initiation location of the specimen was on the edge of the contact area near the lower grip, and the crack propagation direction was almost perpendicular to the moving direction, which is shown in Figure 16. 

**Figure 17:**
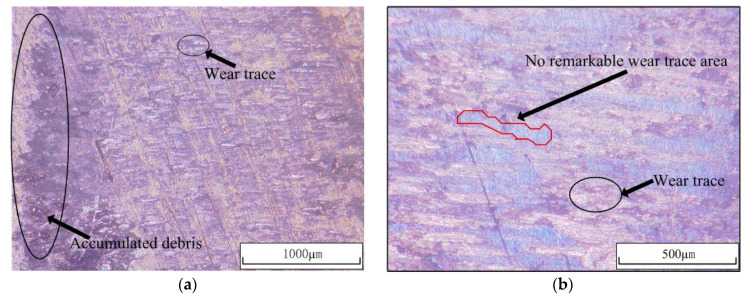

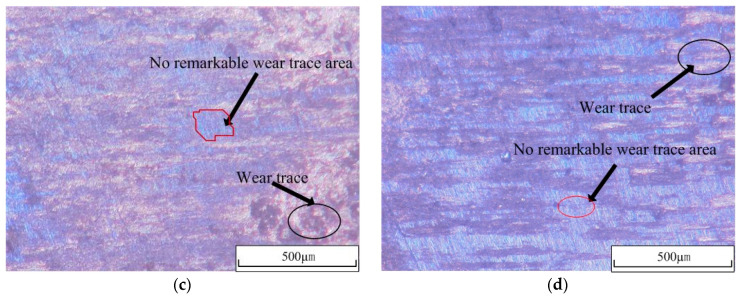
Pictures of the typical wear surfaces of the specimens and pads. (**a**) Wear trace and accumulated debris of untreated specimen. (**b**) Wear trace under laser power density of 3.2 GW/cm^2^. (**c**) Wear trace under laser power density of 4.8 GW/cm^2^. (**d**) Wear trace under laser power density of 6.4 GW/cm^2^.

The typical wear surfaces of the fretting pads were taken with an optical microscope (OM) under the same load conditions and different laser power densities are shown in Figure 17. It is clear to see that because of the fretting phenomenon between the specimen and pads, the debris is accumulated on the edge of the contact area in Figure 17a. It is also seen that because of the larger surface roughness caused by LSP impact, there are no remarkable wear traces in some parts of the LSP samples’ sliding area, which is similar to Kevin’s research [19].

**Figure 18:**
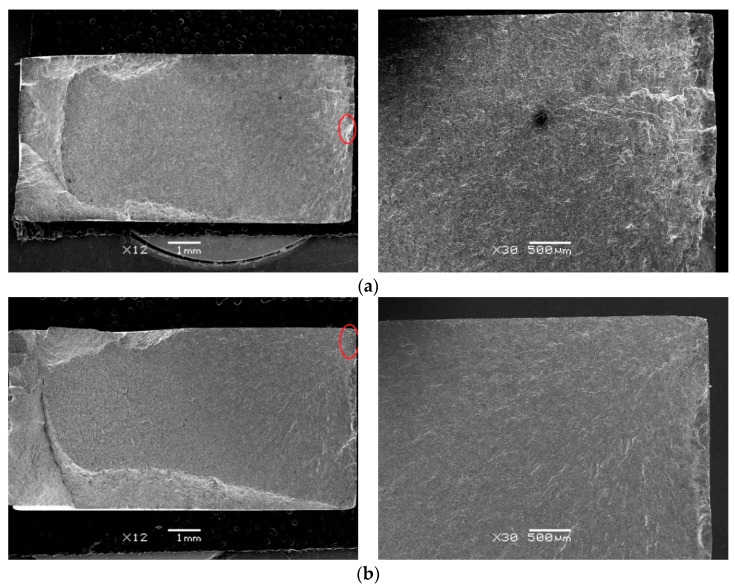

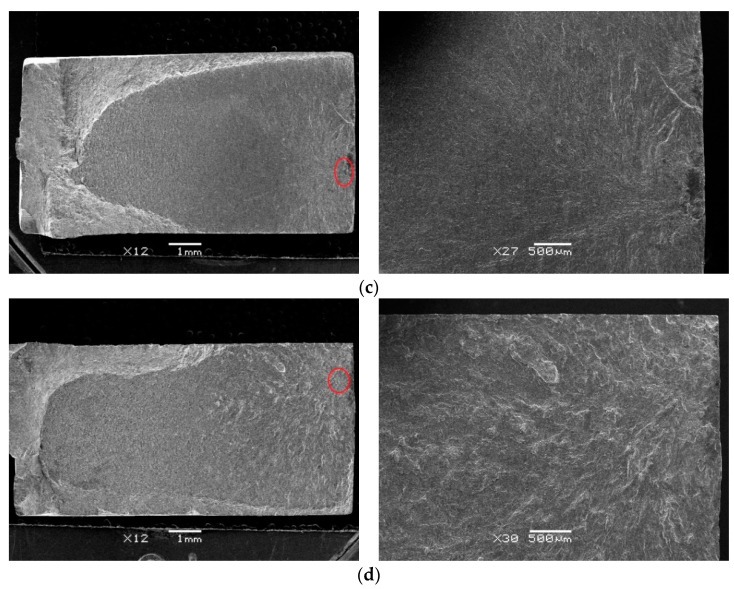
Typical optical microscope (OM) micrographs of the fractures of the specimens. (**a**) Untreated specimen. (**b**) Specimen under laser power density of 3.2 GW/cm^2^. (**c**) Specimen under laser power density of 4.8 GW/cm^2^. (**d**) Specimen under laser power density of 6.4 GW/cm^2^.

Figure 18 shows the typical OM micrographs of the fractures of the specimens for the untreated sample and the LSP samples with different laser power densities, and the main crack source is circled in these pictures. It is found that the fatigue crack source of the untreated specimen and the LSP specimen with 3.2 GW/cm^2^ laser power density are at the surface of the contact area. However, the fatigue crack source of the LPS specimens with 4.8 GW/cm^2^ and 6.4 GW/cm^2^ laser power density are at the subsurface or in the interior of the specimens. This phenomenon shows that the surface compressive residual stresses improve the fatigue resistance of the surface, and lead to the transfer of the crack initiation position to the interior of the specimen.

**Figure 19:**
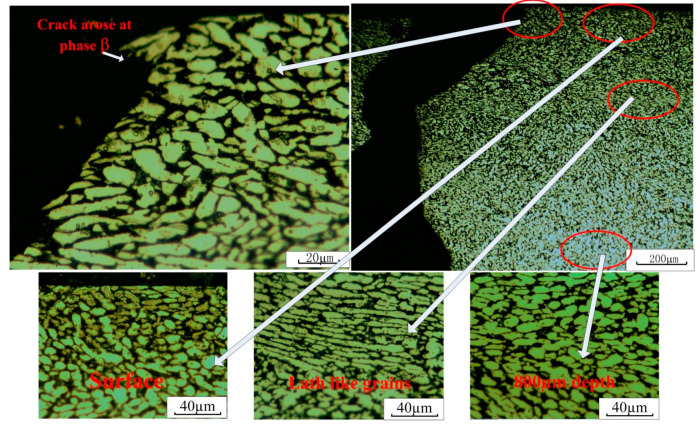
The metallographic of a depth of the main specimen after LSP (6.4 GW/cm^2^).

The metallographic of a depth of the main specimen, which is near the crack, after laser shock peening (6.4 GW/cm^2^) is shown in Figure 19. The formulation of the corrosive agent is as follows: HF:HNO_3_:H_2_O = 1:3:15, and the corrosion time is 20 s.

From Figure 19, it can be seen that compared with the grains under the depth of 800 μm, the grain size of the surface layer is smaller due to LSP. The average grain size of the surface layer is about 6.8 μm, while the average grain size of the 800 microns depth is about 13.7 μm. It is also found that due to the effect of LSP, some grains of the specimen are extruded into a lath like state with the grain thickness of about 3.5 μm. The crack arises due to the location of phases β, and passes through the grains (both α and β) in the process of propagation.

## 4. Conclusions

1. In this paper, the TC11 specimens were under laser shock peening with three different laser power densities of 3.2 GW/cm^2^, 4.8 GW/cm^2^ and 6.4 GW/cm^2^. The surface topography of the untreated and LSP samples were carried out by using a non-contact 3D optical profilometer, and it showed that with the increase of the laser power density, the surface roughness also increased, which meant a worse surface condition.
2. A proto-LXRD X-ray diffractometer was used to measure the surface residual stresses, and the result showed that the laser power density of 4.8 GW/cm^2^ had the best effect with the introduction of compressive residual stresses of −650 Mpa, which meant the plastic deformation of the TC11 surface had reached saturation. The excessive increase of the laser power density would cause the peak pressure of the shock wave to be too large, which reduced the surface quality and induced tensile stress on the surface.
3. A specialized fretting pad fixture and fretting fatigue test rig were used to measure the initiation lives of the fretting fatigue crack. With the comparison between untreated and LSP specimens, it was found that the fretting fatigue life of LSP was significantly improved between 2 and 4 times. In addition, the 4.8 GW/cm^2^ power density had the best effect on the improvement of fatigue life, although the average surface residual stress was similar at the power density of 4.8 GW/cm^2^ and 6.4 GW/cm^2^, the high power density caused the bigger surface damage in the material, which led to the reduction of fatigue life.
4. The OM micrographs of the fractures of the specimens showed that with the increase of laser power density, the source of crack initiation was gradually transferred from the surface to interior of the specimen, which meant that the introduction of LSP improved the surface strength and reduced the surface damage of the specimen, and the fretting effect did not occupy the dominant position in the process of crack initiation. The metallographic of the main specimen showed that the crack arose due to the location of phases β, and passed through the grains (both α and β) in the process of propagation.

## Figures and Tables

**Table 1:** Chemical composition of titanium alloy.

Composition	Al	Mo	Zr	Si	Fe	Ti
Percent (wt./%)	6.40	3.57	1.63	0.25	0.13	Bal

**Table 2:** Material parameters for TC11 at room temperature.

E (MPa)	ν	$\sigma_{-1}\;\left(MPa\right)$	$\sigma_{b}\;\left(MPa\right)$	$\sigma_{0.2\;}\left(MPa\right)$
120,000	0.33	540	1139	866

**Table 3:** Laser shock peening parameters for TC11 specimens.

Wave Length	Pulse Width	Diameter of Laser Spot	Overlapping Rate	Absorbing Layer	Restraint Layer	Laser Power Densities (GW/cm^2^)
1064 nm	20 ns	2 mm	50%	Black tape	Water	3.2, 4.8, 6.4

**Table 4:** Experimental parameters used in fretting fatigue tests.

Series	P (MPa)	Q_max_ (MPa)	Stress Ratio	Laser Power Densities (GW/cm^2^)
1	65.45	400	0.1	No treatment
2	65.45	400	0.1	3.2
3	65.45	400	0.1	4.8
4	65.45	400	0.1	6.4
5	40	400	0.1	No treatment
6	40	400	0.1	3.2
7	40	400	0.1	4.8
8	40	400	0.1	6.4

**Table 5:** Surface roughness of the main specimens with different laser power densities.

Laser Power Density/(GW/cm^2^)	0	3.2	4.8	6.4
Roughness/μm	0.94	1.10	1.28	1.52

**Table 6:** Results of the surface compressive residual stresses.

*I*_0_/(GW/cm^2^)	Residual Stress/(MPa)	Point 3	Point 2	Point 1	Point 4	Point 5
3.2	σ_x_	−138	−521	−563	−559	−121
	σ_y_	−121	−488	−601	−589	−135
4.8	σ_x_	−171	−676	−671	−670	−168
	σ_y_	−161	−639	−630	−619	−164
6.4	σ_x_	−132	−674	−735	−633	−130
	σ_y_	−90	−569	−696	−561	−99

**Table 7:** Experimental fretting fatigue initiation life of TC11 with different laser power densities.

Load	Series 1 (No Treatment)		Series 2 (*I*_0_ = 3.2 GW/cm^2^)	
	Life (Cycles)	Average Life (Cycles)	Life (Cycles)	Average Life (Cycles)
*P* = 65.45 MPa, *Q_max_* = 400 MPa	105,223	88,863	152,527	198,752
	82,750		237,018	
	78,616		206,710	
Load	Series 3 (*I*_0_ = 4.8 GW/cm^2^)		Series 4 (*I*_0_ = 6.4 GW/cm^2^)	
*P* = 65.45 MPa, *Q_max_* = 400 MPa	357,900	323,652	195,221 18,823	192,022
	333,157			
	279,899			
Load	Series 5 (No treatment)		Series 6 (*I*_0_ = 3.2 GW/cm^2^)	
*P* = 40 MPa, *Q_max_* = 400 MPa	133,233	106,599	>400,000	-
	79,450			
	104,500 109,214			
Load	Series 7 (*I*_0_ = 4.8 GW/cm^2^)		Series 8 (*I*_0_ = 6.4 GW/cm^2^)	
*P* = 40 MPa, *Q_max_* = 400 MPa	>430,000	-	282,828	282,828

## References

[1] Hills D.A. (1994). Mechanics of fretting fatigue. Wear.

[2] Golden P.J., Hutson A., Sundaram V., Arps J.H. (2007). Effect of surface treatments on fretting fatigue of Ti–6Al–4V. Int. J. Fatigue.

[3] Nicholas T. (1999). Critical issues in high cycle fatigue. Int. J. Fatigue.

[4] Shen M., Peng J., Zheng J. (2010). Study and Development of Fretting Fatigue. J. Mater. Eng..

[5] Golden P.J., Shepard M.J. (2007). Life prediction of fretting fatigue with advanced surface treatments. Mater. Sci. Eng. A.

[6] Liu D., Tang B., Zhu X., Chen H., He J., Celis J.P. (1999). Improvement of the fretting fatigue and fretting wear of Ti6Al4V by duplex surface modification. Surf. Coat. Technol..

[7] Vantadori S., Valeo J.V., Zanichelli A. (2020). Fretting fatigue and shot peening: A multiaxial fatigue criterion including residual stress relaxation. Tribol. Int..

[8] Liu X., Liu J., Zuo Z., Zhang H. (2019). Effects of Shot Peening on Fretting Fatigue Crack Initiation Behavior. Materials.

[9] Martín V., Vázquez J., Navarro C., Domínguez J. (2020). Effect of shot peening residual stresses and surface roughness on fretting fatigue strength of Al 7075-T651. Tribol. Int..

[10] Zhang H., Yang X., Cui H., Wen W. (2019). Study on the Effect of Laser Quenching on Fretting Fatigue Life. Metals.

[11] Srinivasan S., Garcia D.B., Gean M.C., Murthy H., Farris T.N. (2009). Fretting fatigue of laser shock peened Ti–6Al–4V. Tribol. Int..

[12] Nie X., He W., Zang S., Wang X., Zhao J. (2014). Effect study and application to improve high cycle fatigue resistance of TC11 titanium alloy by laser shock peening with multiple impacts. Surf. Coat. Technol..

[13] Tong Z.P., Ren X.D., Zhou W.F., Adu-Gyamfi S., Chen L., Ye Y.X., Ren Y.P., Dai F.Z., Yang J.D., Li L. (2019). Effect of laser shock peening on wear behaviors of TC11 alloy at elevated temperature. Opt. Laser Technol..

[14] Qiao H., Zhao J. (2013). Analysis and Optimization on Laser Peening Parameters of Tianium Alloy. Acta Optiac Sinaca.

[15] Lei Z.Z., Yinghong L.L., Cheng W.W., Xin Z. (2010). Laser shock peening for LY2 alloy. High. Power Laser Part. Beams.

[16] Ren X.D., Zhang Y.K., Zhou J.Z., Kong D.J. (2006). Thickness optimizing of surface coating for laser shock processing. Heat Treat. Met..

[17] Lee H., Sathish S., Mall S. (2004). Evolution of residual stress under fretting fatigue. J. Mater. Sci..

[18] King A., Steuwer A., Woodward C., Withers P.J. (2006). Effects of fatigue and fretting on residual stresses introduced by laser shock peening. Mater. Sci. Eng. A.

[19] Liu K.K., Hill M.R. (2009). The effects of laser peening and shot peening on fretting fatigue in Ti–6Al–4V coupons. Tribol. Int..

[20] Qiao H., Hu X., Zhao J., Wu J., Sun B., Lu Y., Guo Y. (2019). Influence Parameters and Development Application of Laser Shock Processing. Surf. Technol..

[21] Fabbro R., Fournier J., Ballard P., Devaux D., Virmont J. (1990). Physical study of laser-produced plasma in confined geometry. J. Appl. Phys..

[22] Fabbro R., Peyre P., Berthe L., Scherpereel X. (1998). Physics and applications of laser-shock processing. J. Laser Appl..

[23] Deng Z.H., Liu Q.B., Xu P., Yao Z.H. (2018). Corrosion resistance and mechanism of metallic surface processed by square-spot laser shock peening. J. Mater. Eng..

[24] Jiang Y.F., Ji B., Gan X.D., Hua C., Li X., Zhu H. (2018). Study on the effect of laser peening with different power densities on fatigue life of fastener hole. Opt. Laser Technol..

[25] Hu Y.X. (2008). Research on the Numerical Simulation and Impact Effects of Laser Shock Processing. Ph.D. Thesis.

[26] Peyre P., Fabbro R., Merrien P., Lieurade H.P. (1996). Laser shock processing of aluminium alloys. Application to high cycle fatigue behaviour. Mater. Sci. Eng. A.

[27] Li B.M., Liu X.M., Zhang H.H., Liu F. (2017). Numerical simulation of laser shock processing in 2024 aluminum alloy. Appl. Laser.

[28] Hua G., Jiang S., Cao Y., Zhou D., Chen H. (2017). Numerical simulation of residual stress hole on 7050 aluminum alloy under laser shock. Heat Treat. Met..

[29] Sun C.W. (2002). The Effect of Laser Irradiation.

[30] Aeronautical Manufacture Engineering Committee (1997). Aeronautical Manufacture Engineering Handbook.

